# Synthesis of heteroleptic [Sr(ddemap)(tmhd)]_2_ and its use in atomic layer deposition of low carbon SrO thin films

**DOI:** 10.1039/d5ra08373g

**Published:** 2026-01-21

**Authors:** Yeji Lee, Chanwoo Park, Sangyeon Jeong, Daeun Lim, Jonghyun Kim, Hyeongjun Kim, Eun A Kim, Seong-Yong Cho, Hyobin Yoo, Bo Keun Park, Teak-Mo Chung, Woongkyu Lee

**Affiliations:** a Department of Electrical Engineering, Myongji University Yongin 17558 Republic of Korea; b Thin Film Materials Research Center, Korea Research Institute of Chemical Technology Daejeon 67447 Republic of Korea tmchung@krict.re.kr; c Department of Materials Science and Engineering, Soongsil University Seoul 07040 Republic of Korea woong@ssu.ac.kr; d Department of Photonics and Nanoelectronics, HYU-KITECH Joint Department, Hanyang University Ansan 15588 Korea; e Department of Materials Science and Engineering, Research Institute of Advanced Materials, Seoul National University Seoul 08826 Republic of Korea

## Abstract

New strontium heteroleptic complexes were synthesized by the substitution reaction of the bis(trimethylsilyl)amide of Sr(btsa)_2_·2DME with aminoalkoxide and β-diketonate ligands (btsa = bis(trimethylsilyl)amide, DME = 1,2-dimethoxyethane). Three compounds, [Sr(ddemap)(tmhd)]_2_ (1), [Sr(ddemmp)(tmhd)] (2), and [Sr(ddemamb)(tmhd)]_2_ (3), were obtained as precursors for SrO growth (ddemap = 1-(dimethylamino)-5-((2-(dimethylamino)ethyl)(methyl)amino)pentan-3-ol, ddemmp = 1-(dimethylamino)-5-((2-(dimethylamino)ethyl)(methyl)amino)-3-methylpentan-3-ol, ddemamb = 1-(dimethylamino)-2-(((2-(dimethylamino)ethyl)(methyl)amino)methyl)butan-2-ol). In single crystal X-ray crystallography, complex 1 showed dimeric structure with µ_2_-O bonds of ddemap ligand. Complexes 1 and 2 displayed high volatility and can be sublimed under reduced pressure (0.7 torr) at 150 °C. Accordingly, complex 1 was used as an atomic layer deposition (ALD) precursor for synthesis of SrO thin film at a high temperature of 370 °C. With O_3_ as the oxygen source, typical ALD growth behavior was obtained with controllable initial growth and uniformity. The as-deposited SrO film reacted with carbon in the air and formed SrCO_3_ with high crystallinity and poor surface morphology. However, the Al_2_O_3_ capping layer induced a smooth amorphous SrO film with little carbon or nitrogen impurities, which indicated the high purity of as-grown SrO film with no carbonate phase formation by the novel SrO ALD process.

## Abstract 2

New heteroleptic Sr complexes were synthesized and evaluated as ALD precursors. [Sr(ddemap)(tmhd)]_2_ enabled self-limited SrO growth at 370 °C with O_3_. Al_2_O_3_ capping effectively suppressed carbonate formation, yielding smooth, high-purity SrO films.

## Graphical abstract

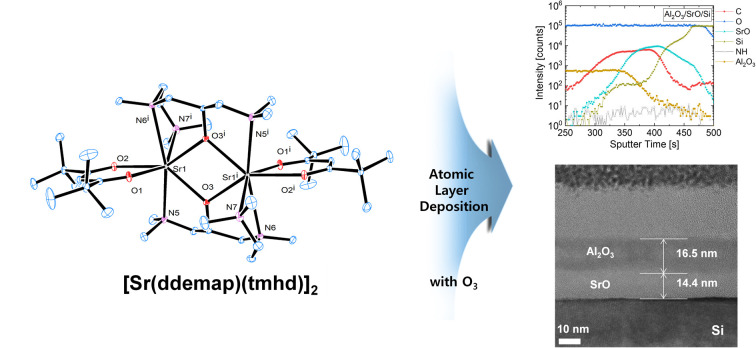

## 1. Introduction

The alkaline-earth metals have been widely used in various technological applications such as thin films,^1–4^ ferroelectrics,^3,5–7^ nonlinear optical materials,^8,9^ and organic synthesis.^10,11^ In particular, strontium complexes play a key role in the semiconductor industry, as it could be used to fabricate thin films containing metal oxides.^1–4^ The SrO film plays an important role as a passivation layer to prevent Si oxidation as well as a barrier to silicide formation during high-k gate oxide deposition.^12,13^ Furthermore, SrO is a key material involved in the growth of perovskite SrTiO_3_, a dielectric material with a very high permittivity (∼300 in bulk), for next-generation dynamic random access memory (DRAM) capacitors.^14–20^ Considering the required large area uniformity and extreme three-dimensional structure of the capacitors, atomic layer deposition (ALD) appears to be the only feasible thin film growth technique.^21–26^ It can fulfill the stringent requirements of conformality in the DRAM capacitors by the alternate supply of the precursor and reactant. The saturation of the surface reactions could be achieved in the ALD process by a self-limited reaction mechanism. Since the SrTiO_3_ ALD process consists of subcycles of SrO and TiO_2_, the optimization of each consisting subcycle ALD and adopting precursors with excellent volatility and thermal stability are crucial.

However, developing new strontium ALD precursors can be challenging due to their tendency to form oligomeric complexes with improper volatility, low thermal stability, and poor reactivity to surface active sites, which is caused by their large radius, small charge, and high coordination number.^9^ As a result, only a few Sr precursors and SrO ALD processes have been reported with excellent physical and chemical properties and growth behavior. Recently, Cp-based materials such as (Sr(^i^Pr_3_C_5_H_2_)_2_ ^27^ and Sr(^*t*^Bu_3_C_5_H_2_)_2_)^28^ have been used as most of the available precursors in SrTiO_3_ ALDs. But there is a problem that strontium complexes containing Cp-based ligands are extremely sensitive to air (oxygen) and moisture,^29^ and SrCO_3_ films were grown instead of SrO or show anomalous overgrowth in the early growth stages of ALD.

This abnormal initial excess growth behavior is mainly due to the high reducing power of the Cp-based Sr precursor or the high oxidizing power of a reactant such as ozone. It caused problems of a large amount of impurities in the film and the degradation of the electrical characteristics of the capacitor. The formation of SrCO_3_ by insufficient ligand exchange reaction during the shortened initial growth stage or by reaction with carbon in the air is a crucial hindrance to the subsequent crystallization of SrTiO_3_ thin films.^17,30,31^ In order to suppress the initial overgrowth of SrO, a trial was conducted in which a 3-nm-thick TiO_2_ or a 1-nm-thick Al_2_O_3_ was applied to prevent the reaction with the highly reactive bottom layer. Lee *et al.* reported that the SrTiO_3_ thin film with a blocking layer exhibited completely linear growth behavior with reduced carbonate formation.^32^ Additionally, the introduction of more reactive Cp-based Ti precursors has resulted in improvement of SrTiO_3_ thin film growth behavior. Ti(Me_5_Cp)(OMe)_3_ served to countervail the excessively strong reactivity of the Cp-based Sr precursor, enabling the growth of a perovskite SrTiO_3_ thin film with no reaction-blocking layer.^33^ In addition, [Sr(demamp)(tmhd)]_2_ precursors having a lower, yet high enough, reactivity than Sr(iPr_3_Cp)_2_ were introduced to restrict chemical-vapor-deposition-like growth behavior.^34^

**Fig. 1:**
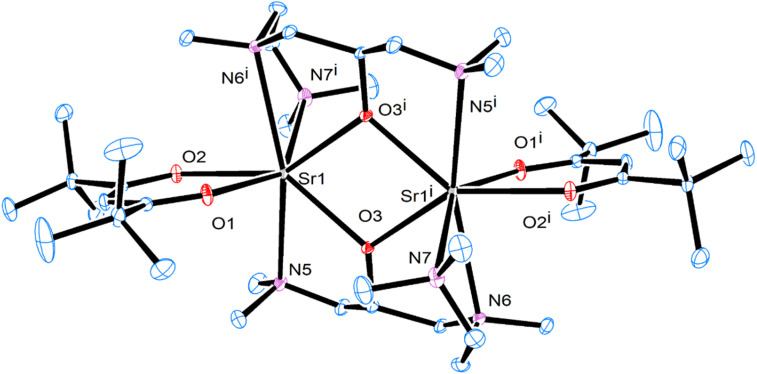
Crystal structure of [Sr(ddemap)(tmhd)]_2_ (1). Thermal ellipsoids are drawn at the 30% probability level.

Based on our previous studies, further research on heteroleptic Sr ALD precursors using aminoalkoxide and β-diketonate ligands was conducted in this work. Highly coordinating aminoalkoxide ligands, such as those in [Sr(tmtad)(btsa)]_2_ ^35^ and [Sr(tmtad)(tmhd)]_2_,^35^ often contain non-coordinated donor groups, which reduce volatility and destabilize the ALD process. In contrast, precursors with lower coordination numbers, including [Sr(bdeamp)(hfac)]_3_,^36^ [Sr(dadamb)(hfac)]_3_,^36^ and [Sr_3_(dadamb)_4_(tmhd)_2_],^36^ tend to form oligomeric species, leading to low vapor pressure and limited ALD performance. In this work, a heteroleptic Sr ALD precursor was designed using a four-coordinate aminoalkoxide ligand together with a β-diketonate ligand. This design forms a fully saturated Sr center without non-bonding groups or oligomerization. The complex [Sr(ddemap)(tmhd)]_2_ (Fig. 1) was synthesized *via* an *in situ* reaction of strontium bis(trimethylsilyl)amide [Sr(btsa)_2_·2DME] and showed good volatility and stable ALD behavior for SrO thin-film deposition and this complex was used as an ALD precursor for SrO thin films. With O_3_ as an oxygen source, smooth and amorphous SrO thin films with low impurity concentration and uniform morphology was successfully deposited by the novel Sr precursor. In addition, the formation and suppression of SrCO_3_ phase were investigated by comparing the surface and bulk regions, and by adopting Al_2_O_3_ capping layer.

## 2. Experimental

### 2.1 General remarks

Nuclear magnetic resonance (NMR) spectra were recorded with a Bruker 500 MHz spectrometer (^1^H) and a Bruker 500 MHz spectrometer (^13^C) with C_6_D_6_ as the solvent and reference. Infrared (IR) spectra were obtained using a Nicolet Nexus FTIR spectrophotometer. Elemental analyses were carried out using a Thermo Scientific OEA Flash 2000 Analyzer. Thermogravimetric analyses (TGA) were conducted using a SETARAM 92–18 TG-DTA instrument with a constant flow of nitrogen (500 mL min^−1^) throughout the experiment in inert condition.

Sr[N(SiMe_3_)]_2_·2DME was prepared following previously reported methods. All reactions were carried out under inert dry conditions in an argon-filled glovebox. Hexane was purified using an Innovative Technology PS-MD-4 solvent purification system. All other chemicals were purchased from Aldrich and Alfa Aesar without further purification. The melting point was measured the Stuart™ SMP40 automatic melting point apparatus.

### 2.2 General procedure for the synthesis of [Sr(L_1–3_)(tmhd)]_2_

A hexane solution (10 mL) of the aminoalcohol ligand L_1–3_ (L_1_ = ddemapH, L_2_ = ddemmpH, and L_3_ = ddemambH) was added dropwise to a solution of Sr[(btsa)]_2_·2DME in hexane (20 mL) at room temperature with constant stirring. After stirring for 20 min at room temperature, 2,2,6,6-tetramethyl-3,5-heptadione (tmhdH) was added to the reaction mixture, which was then stirred for another 15 h at room temperature. Subsequently, it was filtered and the volatiles were removed *in vacuo* to obtain the product as a white solid. X-ray-quality crystals were grown from a saturated solution in toluene at −30 °C.

#### 2.2.1 [Sr(ddemap)(tmhd)]_2_ (1)

Sr(btsa)_2_·2DME (0.59 g, 1.0 mmol) and ddemapH (0.20 g, 1.0 mmol) were used. Yield: 0.38 g (82%). ^1^H NMR (500 MHz, C_6_D_6_): *δ* = 1.33 (s, 18H), 1.84–2.14 (m, br, 4H), 2.22–2.83 (m, br, 4H), 2.12 (s, 3H), 2.17 (s, 6H), 2.41 (s, 6H), 4.21 (m, 1H), 5.81 (m, 1H) ppm. ^13^C NMR (125 MHz, C_6_D_6_): *δ* = 29.2, 41.1, 45.7, 51.9, 58.2, 67.8, 70.1, 87.5, 197.4 ppm. FT-IR (KBr, cm^−1^) = 2949 (s), 2898 (m), 2820 (s), 2790 (s), 1602 (s), 1576 (m), 1473 (m), 1455 (s), 1419 (s), 1356 (m), 1182 (m), 1144 (m), 1039 (w), 863 (w). C_42_H_86_N_6_O_6_Sr_2_ (946.40) calcd.: C, 53.30; H, 9.16; N, 8.88. Found: C, 52.73; H, 9.22; N, 8.96.

#### 2.2.2 [Sr(ddemmp)(tmhd)]_2_ (2)

Sr(btsa)_2_·2DME (0.59 g, 1.0 mmol) and ddemmpH (0.22 g, 1.0 mmol) were used. Yield: 0.41 g (85%). M. P. > 200 °C. ^1^H NMR (500 MHz, C_6_D_6_): *δ* = 1.32 (s, 18H), 2.04–2.40 (m, br, 8H), 2.23 (s, 3H), 2.24 (s, 6H), 2.50 (s, 6H), 5.81 (s, 1H) ppm. ^13^C NMR (125 MHz, C_6_D_6_): *δ* = 29.2, 34.6, 41.1, 46.5, 46.6, 48.9, 58.0, 58.6, 71.4, 73.9, 74.6, 87.8, 197.4 ppm. FT-IR (KBr, cm^−1^) = 2949 (s), 2898 (m), 2861 (m), 2820 (m), 2790 (m), 1602 (s), 1576 (m), 1505 (m), 1473 (m), 1455 (s), 1419 (s), 1356 (m), 1182 (m), 1144 (m), 1039 (w), 960 (w), 863 (w). C_44_H_90_N_6_O_6_Sr_2_ (974.46) calcd.: C, 53.77; H, 9.23; N, 8.75. Found: C, 53.98; H, 9.68; N, 8.58.

#### 2.2.3 [Sr(ddemamb)(tmhd)]_2_ (3)

Sr(btsa)_2_·2DME (0.59 g, 1.0 mmol) and ddemambH (0.23 g, 1.0 mmol) were used. Yield: 0.38 g (75%). M. P. > 200 °C. ^1^H NMR (500 MHz, C_6_D_6_): *δ* = 1.32 (s, 18H), 1.38 (s, 3H), 1.61–1.82 (m, br, 2H), 2.05 (m, 2H), 2.10–2.40 (m, br, 6H), 2.17 (s, 3H), 2.23 (s, 6H), 2.51 (s, 6H), 5.81 (s, 1H) ppm. ^13^C NMR (125 MHz, C_6_D_6_): *δ* = 10.2, 29.2, 39.3, 41.1, 45.7, 46.5, 48.8, 58.0, 58.5, 66.4, 71.2, 75.5, 87.8, 197.3 ppm. FT-IR (KBr, cm^−1^) = 2961 (s), 2947 (s), 2862 (m), 2826 (m), 2786 (m), 1600 (s), 1503 (m), 1455 (m), 1419 (s), 1354 (m), 1183 (w), 1128 (w), 1023 (w), 969 (w), 861 (w). C_46_H_94_N_6_O_6_Sr_2_ (1002.51) calcd.: C, 55.11; H, 9.45; N, 8.38. Found: C, 54.24; H, 9.75; N, 8.23.

### 2.3 Crystallography of single crystals

Single crystal of 1 was grown from a saturated solution in toluene at −30 °C. Specimens of suitable size and quality were coated with Paratone oil and mounted onto a glass capillary. Reflection data were collected on a Bruker Apex II-CCD area detector diffractometer with graphite monochromatized Mo–Kα radiation (*λ* = 0.71073 Å). The cell parameters were determined and refined by the SMART program, and data reduction was performed using the SAINT software. The data were corrected for Lorentz and polarization effects, and an empirical absorption correction was applied using the SADABS program. The structures were solved by direct methods, and all non-hydrogen atoms were subjected to anisotropic refinement by full-matrix least-squares on F^2^ using the SHELXTL/PC package. Hydrogen atoms were placed at their geometrically calculated positions and refined based on the corresponding carbon atoms with isotropic thermal parameters. The supplementary crystallographic data for this paper can be found in the Cambridge Crystallographic Data Centre (CCDC): 2239663 (complex 1). These data can be obtained free of charge from the CCDC.

### 2.4 Thin film growth

The SrO thin films were deposited in a traveling wave-type ALD reactor (CN-1 Co., Atomic Basic) for a 6-inch diameter single wafer. The newly developed [Sr(ddemap)(tmhd)]_2_ and high density (200 g m^−3^) O_3_ were used as the Sr-precursor and oxygen source, respectively. The working pressure was 1.2 torr. The deposition temperature was set at 370 °C. The typical SrO ALD sequence consisted of Sr-precursor injection, Ar purge, O_3_ injection, and Ar purge. Al_2_O_3_ was employed as a capping layer to protect the deposited SrO film. Al_2_O_3_ ALD process used trimethylaluminum (TMA) and O_3_ as Al-precursor and oxygen source, respectively, consisting of TMA injection (0.1 s), Ar purge (30 s), O_3_ injection (0.5 s), and Ar purge (30 s).

### 2.5 Thin film characterization

The physical thickness of the SrO and Al_2_O_3_ thin films was measured by ellipsometry (Gaertner Scientific Corporation, L116D). The carbon incorporation in the film and the depth profile of the films were confirmed by time-of-flight secondary ion mass spectrometry (ToF-SIMS, ION-TOF, TOFSIMS.5). The chemical binding states of the thin films were determined by X-ray photoelectron spectroscopy (XPS, Thermo Fisher Scientific, NEXSA). A cross-section transmission electron microscopy (TEM) specimen was prepared using a focused ion beam (Helios G4, Thermo Scientific) technique for the crystallinity and interface analysis of Al_2_O_3_/SrO/Si. High-resolution TEM (HRTEM) lattice imaging was conducted using a 200 kV field-emission TEM (JEM 2100F, Jeol) equipped with a CMOS detector (OneView, Gatan). Crystallinity was investigated by glancing angle mode X-ray diffraction (GAXRD, Rigaku, D/MAX2500). The film surface morphology and root-mean-square (RMS) roughness were investigated using field-emission scanning electron microscopy (FE-SEM, Hitachi, S-4800) and atomic force microscopy (AFM, PSIA, XE150).

## 3. Results and discussion

The volatile heteroleptic complex [Sr(tmtad)(tmhd)]_2_ which has tmtad and tmhd as coordinating bulky ligands to saturate the metal center has been recently reported. In the previous study, these complexes showed a dimeric structure with non-bonding amine groups in the crystal structure and their volatile properties which encouraged further research toward development of strontium precursor with improved properties. The simple preparation of [Sr(L_1–3_)(tmhd)]_2_ complexes was conducted by controlled substitution reaction using strontium bis(trimethylsilyl)amide [Sr(btsa)_2_·2DME] and appropriate ligands (tmhdH and L_1–3_) to yield the desired product [Sr(ddemap)(tmhd)]_2_ (1), [Sr(ddemmp)(tmhd)]_2_ (2), and [Sr(ddemamb)(tmhd)]_2_ (3) as shown in Fig. 1. The L_1–3_ ligands were originally intended to fully saturate the metal center without a non-coordinating amine group.

The ^1^H-NMR spectra of complexes 1–3 in the benzene-d_6_ solution showed a downfield shift of the two amino groups (–N(CH_3_)_2_) compared to the free ligands (CCH_2_N(CH_3_)_2_) and the absence of –btsa peak (Fig. S1–6). FT-IR spectra of all complexes displayed the absence of Si–CH_3_ rocking vibration and –NH peaks from btsa group but showed peaks for C=O stretching in coordinated β-diketones at 1602 cm^−1^, 1602 cm^−1^, and 1600 cm^−1^, respectively, showing that the reaction proceeded successfully (Fig. S7–9).

**Fig. 2:**
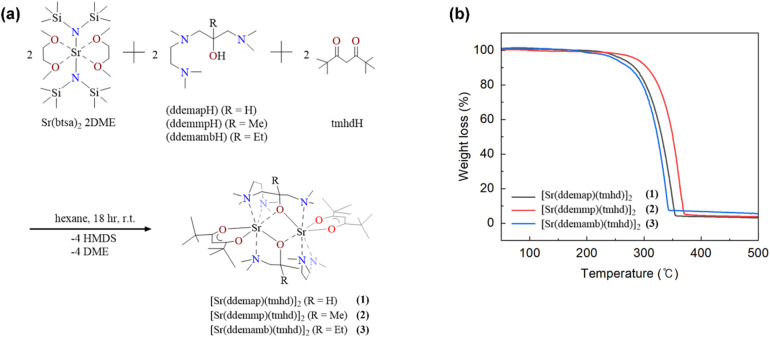
(a) Synthetic scheme and (b) TGA plots of complexes 1–3.

All complexes were obtained as white crystalline powder, where complexes 1–3 showed very good yields and were purified by recrystallization from saturated solution. The single crystal X-ray study reveals that complex 1 crystallizes in the monoclinic space group and exists as dimers where each of the strontium metal centers is bonded to one ddemap and one β-diketonate ligand (Fig. 2(a)). The crystal structure of 1 displayed a fully saturated metal center with three nitrogen atoms from two tmtad ligands and four oxygen atoms from two tmtad and one tmhd ligands where the crystal structure showed distorted capped trigonal prismatic structure. This complex was formed by µ_2_–O bridging where the distance between the two metal centers in 1 (3.888(3) Å) is longer than those of the previously reported oxygen-bridged complexes [Sr(demamp)(tmhd)]_2_ and [Sr(etmtad)(tmhd)]_2_ (Sr⋯Sr = 3.7119(6) and 3.842(4)).^35^ The bridging angles (Sr–O–Sr) in 1 were 106.47° which is comparable to [Sr(etmtad)(tmhd)]_2_ (110.81°). The bond lengths of Sr–N ranged from 2.790–2.844 Å and those of Sr to O ranged from 2.419–2.496 Å (Tables S1 and S2).

TGA of compounds 1 to 3 were conducted from 25 °C to 500 °C. Prior to analysis, samplings were carried out in an Ar-filled glove box and data were collected under a constant flow of N_2_ to avoid any possible contamination by air. Complexes 1–3 displayed clean one-step curves with 99.25%, 96.44%, and 95.01% mass losses and 2.75%, 3.56%, and 4.99% residual mass, respectively, in the 200–350 °C region where the increase in molecular weight should influence the thermal volatility (Fig. 2(b)). All complexes showed high volatility and a low amount of the non-volatile residues indicating that they have high potential as ALD or CVD precursors for fabricating metal oxide thin film. In order to confirm the volatility of complexes 1–3, sublimation tests were carried out under 0.7 torr. Compounds 1 and 2 exhibited good volatile character and sublimed at 150 °C whereas compound 3 was decomposed at this condition. Among these complexes, compound 1, [Sr(ddemap)(tmhd)]_2_, with the lower residual mass and higher volatility than other compounds was adopted as ALD precursor for the fabrication of SrO film.

**Fig. 3:**
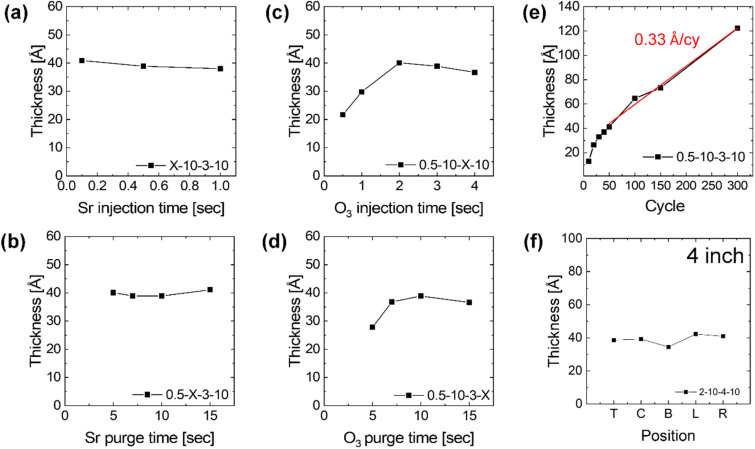
(a–d) Typical saturation behavior of ALD process forming SrO films. The unit process during a cycle is sequential stages of precursor (Sr) injection-Ar (Sr) purge-reactant (O_3_) injection-Ar (O_3_) purge, 40 cycles of ALD process on a silicon substrate at 370 °C. (e) Plot of the thickness change as a function of the cycle, the ALD process times for each step were fixed to 0.1 s–10 s–3 s–10 s. (f) The thickness variation within 4-in wafer.

ALD deposition process of a SrO thin film on Si substrate was developed at 370 °C when [Sr(ddemap)(tmhd)]_2_ and O_3_ were employed as the Sr precursor and oxygen source, respectively. Fig. 3(a–d) shows the typical ALD saturation growth behavior for SrO thin film at 370 °C on Si substrate. Each of Sr-precursor injection, purge, O_3_ injection, and purge step time was increased while other steps were fixed to 0.5, 10, 3, or 10 s in Fig. 3(a–d), respectively. The optimized step times were set to 0.1 s–5 s–3 s–10 s in this study. Fig. 3(a–d) indicates [Sr(ddemap)(tmhd)]_2_ precursor shows self-limited surface reactions with O_3_ in film growth of ALD at 370 °C. Despite the complex dimer structure and solid phase of the novel Sr precursor, the required Sr precursor injection time for the SrO film to reach the ALD saturation level was only 0.1 s. 5 s of Sr purge time using the inert Ar gas was sufficient for the removal of the excessive Sr precursors and byproducts. Fig. 3(e) shows the variations in the film thickness as a function of the deposition cycle number under optimized ALD conditions of injection/purge times. As the number of deposition cycles increased, the deposition amount of SrO thin film increased linearly, resulting in a thin film growth per cycle (GPC) of 0.33 Å per cycle. The slight overgrowth observed during the initial ∼40 cycles is attributed to a higher reactivity of the substrate surface toward the Sr precursor compared to that of the subsequently grown SrO surface. As the substrate becomes progressively covered, the surface reactions transition to film–precursor interactions, and the growth rate converges to the steady-state ALD growth regime. Fig. 3(f) depicts the uniformity characteristics according to various positions within the 4-inch-diameter Si wafer. The thickness of SrO films was ∼4 nm, showing a good uniformity of 94%.

**Fig. 4:**
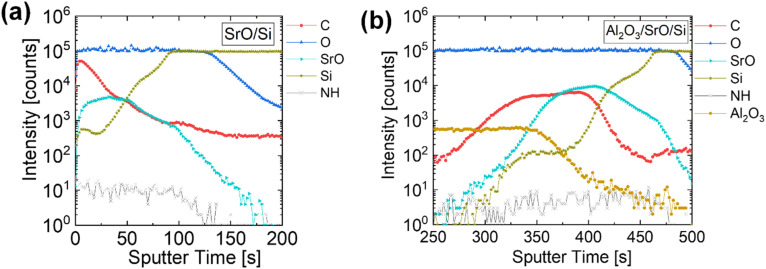
ToF-SIMS depth profiles of (a) 19.0 nm SrO/Si and (b) 16.5 nm Al_2_O_3_/14.4 nm SrO/Si.

**Fig. 5:**
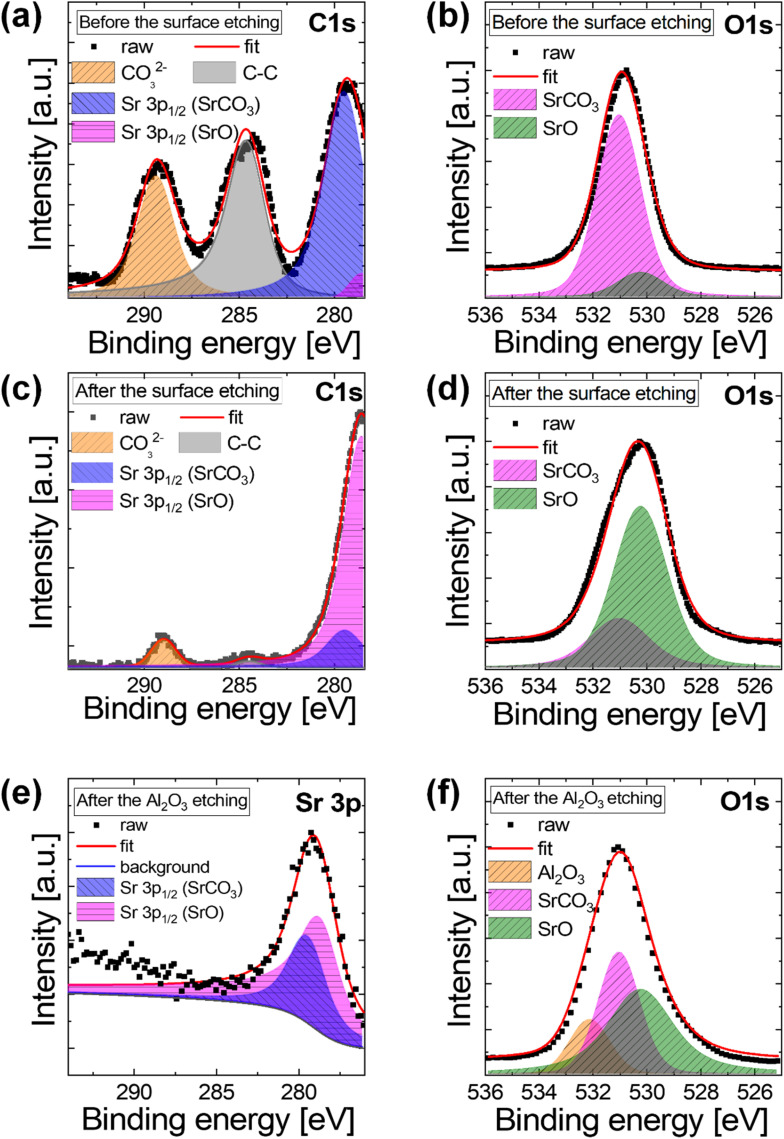
(a–d) XPS spectra of 19 nm SrO film. (a) C 1s and (b) O 1s before the surface etching. (c) C 1s and (d) O 1s after the surface etching. (e and f) XPS spectra of 16.5 nm Al_2_O_3_/14.4 nm SrO after Al_2_O_3_ etching. (e) Sr 3p and (f) O 1s of the interfacial region of Al_2_O_3_/SrO.

The ToF-SIMS depth profiles of a 19 nm SrO film and a 17 nm Al_2_O_3_/14 nm SrO film grown on Si were shown in Fig. 4(a) and (b), respectively. For both samples, the ToF-SIMS depth profile of the SrO film showed a uniform Sr and O composition, and no nitrogen was detected, which is a possible impurity from N containing Sr precursor. In Fig. 4(a), the carbon impurity was concentrated at the top of the SrO film, which is explained by the carbon adsorption by air exposure before chemical analysis. Since SrO is a basic oxide, it has a strong tendency to chemisorb carbon dioxide and water vapor. Comparing the Gibbs free energy at a given temperature, the SrCO_3_ state is thermodynamically more stable than the SrO state.^17^ Therefore, it is inferred that the adsorbed carbon atoms easily react with SrO and form SrCO_3_. The carbon impurity value of the SrO film gradually decreased from the surface of the thin film to the substrate. As will be explained later in Fig. 5, however, despite the reduced carbon impurity in the SrO film, XPS analysis revealed the slight involvement of the SrCO_3_ component in the SrO film. Accordingly, Al_2_O_3_ coating without vacuum break after SrO deposition successfully restricted the air exposure of SrO films and suppressed the carbon concentration in SrCO_3_ in Fig. 4(b). Al_2_O_3_ layer is a well-known capping layer against the penetration of oxygen and moisture. As more than 10 nm Al_2_O_3_ was adopted, the C intensity in ToF-SIMS obviously decreased by ∼10 times. These observations indicate that the dominant source of carbonate formation is associated with post-deposition air exposure. While minor contributions from the ALD process itself cannot be fully excluded, the experimental evidence suggests that carbonate incorporation is primarily surface-driven rather than bulk-induced by precursor chemistry. The ligand exchange reaction successfully took place and carbon or nitrogen in the precursor completely purged out as they were included in the byproducts. Regarding the relatively high carbon contamination at the Al_2_O_3_/SrO interface in Fig. 4(b), another possible cause for the carbon incorporation is TMA adopted for Al_2_O_3_ capping layer ALD. Three –CH_3_ ligands from TMA may contribute to residual carbon at oxide interfaces or within the growing film, particularly during the initial stages of deposition.^37^ Therefore, when the Al_2_O_3_ layer is deposited, the SrO film surface faces methyl groups. The –CH_3_ ligand of the TMA precursor reacts with the surface of the SrO thin film, and carbon impurities may be included at the Al_2_O_3_ and SrO interface. Nevertheless, comparing the SrO film with and without Al_2_O_3_ capping layer, the carbon intensity of the Al_2_O_3_/SrO film decreased, which indicates the carbon contamination by TMA is much lower than by air. Whether the carbon contamination by TMA is severe or not, the developed SrO ALD process itself with [Sr(ddemap)(tmhd)]_2_ and O_3_ offers SrO thin films with high purity.

The chemical status of the single SrO film and Al_2_O_3_/SrO film was examined by XPS. For binding energy calibration of various spectra of XPS data, C–C bonding energy located at 284.5 eV was used as a reference.^33,34,38^Fig. 5(a) is the C 1s spectra before etching the surface of the SrO film in XPS. The C–C bonding (284.5 eV) at the surface region is due to air exposure as mentioned in ToF-SIMS analysis. Owing to the diffusion of carbon, the peak of SrCO_3_ (289.4 eV) appeared strongly in the C 1s spectrum. In the lower energy region corresponding to Sr 3p spectrum, the presence of the Sr 3p_1/2_ peak of SrCO_3_ was obvious at 279.4 eV, and the Sr 3p_1/2_ peak (278.5 eV) of SrO binding energy also appeared. In the O 1s spectrum of Fig. 5(b), the surface of SrO film showed both the SrCO_3_ peak (531 eV) and the SrO peak (530.2 eV).^39^ As in the C 1s spectrum, the O 1s spectrum before surface etching was dominated by the SrCO_3_ component, which accounted for 87.43% of the total O 1s peak area, while the SrO component contributed 12.57%, indicating severe carbonate formation at the air-exposed surface.

Since the carbon contamination by air exposure from the sample surface was critical in Fig. 4, the chemical property inside the film was examined after the surface etching until C–C 1s bonding peak almost disappeared in Fig. 5(c) and (d) for C 1s and O 1s, respectively. The intensity of the SrCO_3_ (289.4 eV) peak was much weaker after surface etching in Fig. 5(c), and the strong SrO peak was observed in the Sr 3p energy regions after the surface etching. Although the area of SrCO_3_ abruptly decreased in the bulk region of the deposited film in C 1s spectra, a sign of SrCO_3_ binding energy in the Sr 3p still remained as a small peak after the surface etching. As shown in Fig. 5(d) after surface etching of the SrO film, the SrCO_3_ peak area fraction was markedly reduced to 26.16%, while the SrO component became dominant, accounting for 73.84% of the O 1s peak area, demonstrating that carbonate species were largely confined to the near-surface region. To investigate the capping layer effect of Al_2_O_3_, the top Al_2_O_3_ capping layer was etched in XPS chamber for Al_2_O_3_/SrO film to examine the chemical properties at the interface of Al_2_O_3_/SrO. After etching the Al_2_O_3_ layer, the C–C bonding of C 1s and the SrCO_3_ peak completely disappeared, and the SrO and SrCO_3_ peaks were observed in Sr 3p energy region as shown in Sr 3p spectra in Fig. 5(e). As explained in the ToF-SIMS results, SrCO_3_ involvement might be attributed to the reaction with the TMA precursor ligand. In the spectrum of O 1s after Al_2_O_3_ etching in Fig. 5(f), Al_2_O_3_ peak was still present indicating adequate etching until interface. In the O 1s spectrum after Al_2_O_3_ etching of the Al_2_O_3_/SrO stack (Fig. 5(f)), both SrO and SrCO_3_ components were observed at the interface, with SrO accounting for 55.63% of the O 1s peak area and SrCO_3_ for 44.37%, indicating substantial suppression of carbonate formation compared to the air-exposed SrO surface. Based on the quantitative XPS analysis, the dominant carbonate contribution is associated with post-deposition air exposure, while the bulk SrO film retains a predominantly oxide character, although minor contributions from the ALD process of SrO (or Al_2_O_3_) itself cannot be fully excluded.

**Fig. 6:**
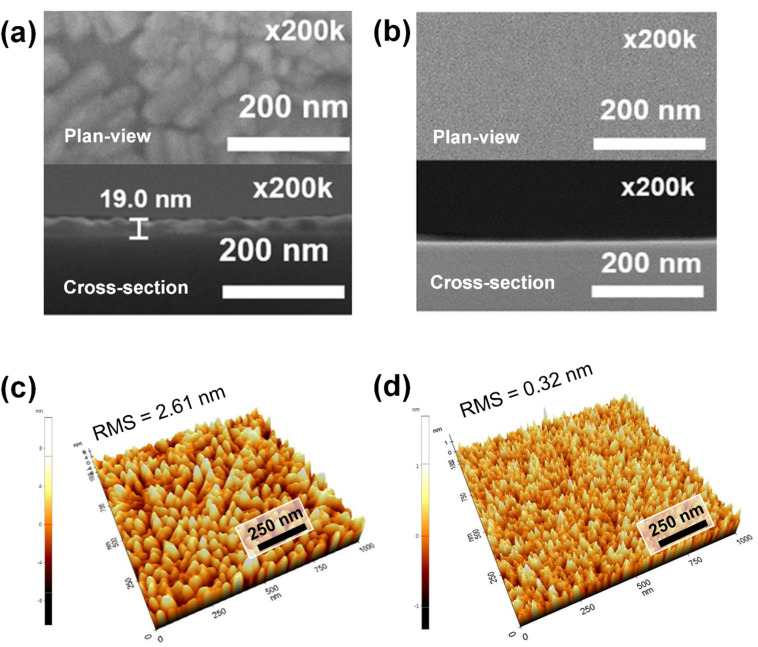
Plan-view and cross-sectional SEM images (a) 19.0 nm SrO film and (b) 16.5 nm Al_2_O_3_/14.4 nm SrO film structure. AFM topography images of (c) 19.0 nm SrO film and (d) 16.5 nm Al_2_O_3_/14.4 nm SrO film structure. The RMS roughness value decreased from 2.61 nm in the as-deposited SrO structure to 0.32 nm with Al_2_O_3_ capping layer.

The surface morphology of the SrO film and Al_2_O_3_/SrO film by plan-view and cross-sectional SEM image was shown in Fig. 6(a) and (b). The as-deposited SrO film showed a quite rough and porous surface morphology in Fig. 6(a). On the other hand, the film of Al_2_O_3_/SrO structure has a highly conformal and smooth surface in Fig. 6(b). Fig. 6(c) and (d) show the AFM topography images of the SrO film and Al_2_O_3_/SrO film, respectively. The RMS roughness of the air-exposed SrO film (2.61 nm) is significantly higher than that of the Al_2_O_3_-capped SrO film (0.32 nm). This difference does not simply reflect the intrinsic smoothness of ALD-grown Al_2_O_3_, but rather highlights the severe surface roughening induced by SrCO_3_ crystallization upon air exposure of SrO. The smooth morphology of the Al_2_O_3_/SrO structure confirms that suppression of carbonate formation is essential for maintaining the intrinsic surface quality of ALD-grown SrO films. The high RMS roughness value of the SrO film is consistent with the rough and porous surface morphology observed in the SEM image.

**Fig. 7:**
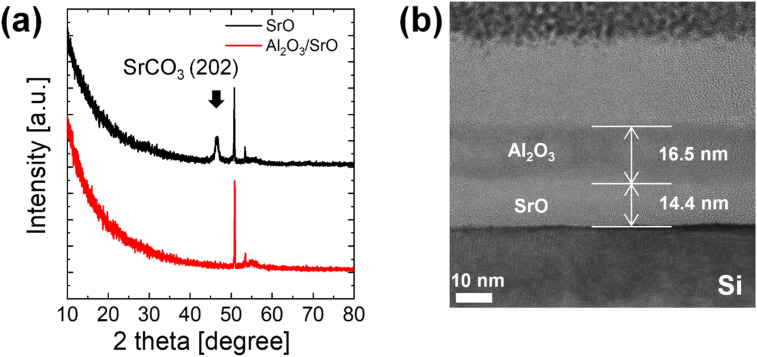
(a) GAXRD of SrO with and without Al_2_O_3_ capping layer (ref. ICDD card no. 00-005-0418). The unmarked peaks correspond to substrate. (b) Cross-sectional HRTEM image of Al_2_O_3_/SrO structure.

The crystallinity was investigated to find out the effect of the involvement of carbon on the crystallinity of the SrO. Fig. 7(a) shows the GAXRD patterns of the as-deposited SrO film and Al_2_O_3_ capped SrO film. While the as-deposited SrO film without the capping layer exhibited a crystalline SrCO_3_ (202) peak at 46.5°, this crystallinity does not originate from the intrinsic ALD-grown SrO film, but rather from post-deposition chemical conversion of SrO to SrCO_3_ upon air exposure. In contrast, the Al_2_O_3_-capped SrO film showed no crystalline peaks, indicating that the *in situ* Al_2_O_3_ layer effectively suppresses ambient-induced carbonate formation and preserves the amorphous nature of the as-grown SrO. The high crystallinity and amorphous nature of the samples correspond well to the surface morphologies in Fig. 6. It should be emphasized that the Al_2_O_3_ capping layer does not modify the intrinsic ALD growth mechanism of SrO, but rather preserves its as-deposited chemical and structural state by preventing post-deposition environmental reactions. In the cross-sectional HRTEM image of the Al_2_O_3_/SrO/Si structure in Fig. 7(b), SrO thin film exhibits an amorphous structure with uniform thickness and sharp interfaces. The formation of a SiO_2_ interfacial layer was not observed at the interface between the SrO film and the Si substrate although high density O_3_ was used during the deposition.

## 4. Conclusions

A new heteroleptic strontium complexes, [Sr(ddemap)(tmhd)]_2_ (1), [Sr(ddemmp)(tmhd)]_2_ (2), and [Sr(ddemamb)(tmhd)]_2_ (3) were synthesized and characterized. All complexes were obtained as white crystalline powders, where complex 1 displayed a dimeric structure with the distorted capped trigonal prismatic geometry in single-crystal X-ray crystallography. These compounds showed a clean one-step TG curve and low non-volatile residue, where compounds 1 and 2 sublimed at 0.7 torr (150 °C). Especially, complex 1 was used to fabricate SrO thin films with O_3_. Typical ALD saturation behaviors were achieved at 370 °C on Si substrates. The as-deposited SrO films reacted with adsorbed carbon by air exposure and resulted in SrCO_3_ crystalline phase with voids and cracks in the film. However, with the aid of Al_2_O_3_ capping layer, it was revealed that smooth amorphous SrO film with little impurity was achievable with the novel Sr-precursor, [Sr(ddemap)(tmhd)]_2_ and O_3_ by ALD. This indicates that when the developed SrO ALD is used at inner part of the device or is used as a sub-ALD process for multicomponent thin film such as SrTiO_3_, SrRuO_3_, or (Ba,Sr)TiO_3_, the grown film could have preferable film property of SrO with Al_2_O_3_ capping layer in this study.

## Author contributions

Yeji Lee: investigation, data curation, formal analysis, conceptualization, visualization, writing – original draft, Chanwoo Park: investigation, data curation, formal analysis, writing – original draft, Sangyeon Jeong: validation, formal analysis, writing – review and editing, Daeun Lim: visualization, validation, Jonghyun Kim: data curation, Hyeongjun Kim: validation, visualization, Eun A Kim: investigation, Seong-Yong Cho: resource, writing – review and editing, Hyobin Yoo: investigation, data curation, Bo Keun Park: methodology, conceptualization, Teak-Mo Chung: methodology, writing – review and editing, funding acquisition, supervision, resources, Woongkyu Lee: conceptualization, methodology, writing – review & editing, supervision, project administration, funding acquisition, resources.

## Conflicts of interest

The authors declare that they have no known competing financial interests or personal relationships that could have appeared to influence the work reported in this paper.

## Supplementary Material

RA-016-D5RA08373G-s001

RA-016-D5RA08373G-s002

## Data Availability

All relevant data supporting this article are included within the manuscript and supplementary information (SI). Supplementary information: detailed experimental characterization data for the synthesized strontium complexes, including ^1^H and ^13^C NMR spectra, FT-IR spectra, and single-crystal X-ray diffraction data (tables of crystallographic parameters, bond lengths, and bond angles). See DOI: https://doi.org/10.1039/d5ra08373g. CCDC 2239663 contains the supplementary crystallographic data for this paper.^40^
