# Correction: Synthesis of heteroleptic [Sr(ddemap)(tmhd)]_2_ and its use in atomic layer deposition of low carbon SrO thin films

**DOI:** 10.1039/d6ra90050j

**Published:** 2026-05-27

**Authors:** Yeji Lee, Chanwoo Park, Sangyeon Jeong, Daeun Lim, Jonghyun Kim, Hyeongjun Kim, Eun A Kim, Seong-Yong Cho, Hyobin Yoo, Bo Keun Park, Taek-Mo Chung, Woongkyu Lee

**Affiliations:** a Department of Electrical Engineering, Myongji University Yongin 17558 Republic of Korea; b Thin Film Materials Research Center, Korea Research Institute of Chemical Technology Daejeon 67447 Republic of Korea tmchung@krict.re.kr; c Department of Materials Science and Engineering, Soongsil University Seoul 07040 Republic of Korea woong@ssu.ac.kr; d Department of Photonics and Nanoelectronics, HYU-KITECH Joint Department, Hanyang University Ansan 15588 Korea; e Department of Materials Science and Engineering, Research Institute of Advanced Materials, Seoul National University Seoul 08826 Republic of Korea

## Abstract

Correction for ‘Synthesis of heteroleptic [Sr(ddemap)(tmhd)]_2_ and its use in atomic layer deposition of low carbon SrO thin films’ by Yeji Lee *et al.*, *RSC Adv.*, 2026, **16**, 4740–4749, DOI: https://doi.org/10.1039/d5ra08373g

The authors regret that the name of one of the authors (Taek-Mo Chung) was shown incorrectly in the original article. The corrected author list is as shown above.

The Royal Society of Chemistry apologises for these errors and any consequent inconvenience to authors and readers.

