# Supplementary material for: Synthesis of heteroleptic [Sr(ddemap)(tmhd)]2 and its use in atomic layer deposition of low carbon SrO thin films
Source: RSC Adv. 2026 Jan 21;16(6):4740–9. doi: 10.1039/d5ra08373g (PMC12822820; doi:10.1039/d5ra08373g)
Supplement: RA-016-D5RA08373G-s001 [file RA-016-D5RA08373G-s001.pdf]

Supplementary Information (SI) for RSC Advances.
This journal is © The Royal Society of Chemistry 2026

Supporting Information

Synthesis of Heteroleptic [Sr(ddmap)(tmhd)]$_2$ and Its Use in Atomic Layer Deposition of Low Carbon SrO Thin Films

Yeji Lee[1#], Chanwoo Park[2#], Sangyeon Jeong[3], Daeun Lim[1,3], Jonghyun Kim[1], Hyeongjun Kim[3], Eun A Kim[4], Seong-Yong Cho[4], Hyobin Yoo[5], Bo Keun Park[2], Teak-Mo Chung[2*], and Woongkyu Lee[1,3]*

[1]Department of Electrical Engineering, Myongji University, Yongin 17558, Republic of Korea

[2]Thin Film Materials Research Center, Korea Research Institute of Chemical Technology, Daejeon 67447, Republic of Korea

[3]Department of Materials Science and Engineering, Soongsil University, Seoul 07040, Republic of Korea

[4]Department of Photonics and Nanoelectronics, HYU-KITECH Joint Department, Hanyang University, Ansan 15588, Korea

[5]Department of Materials Science and Engineering, Research Institute of Advanced Materials, Seoul National University, Seoul 08826, Republic of Korea

[#] Y. Lee and C. Park contributed equally to this work.

[*]Author to whom correspondence should be addressed: W. Lee (woong@ssu.ac.kr), T.-M. Chung (tmchung@krict.re.kr)

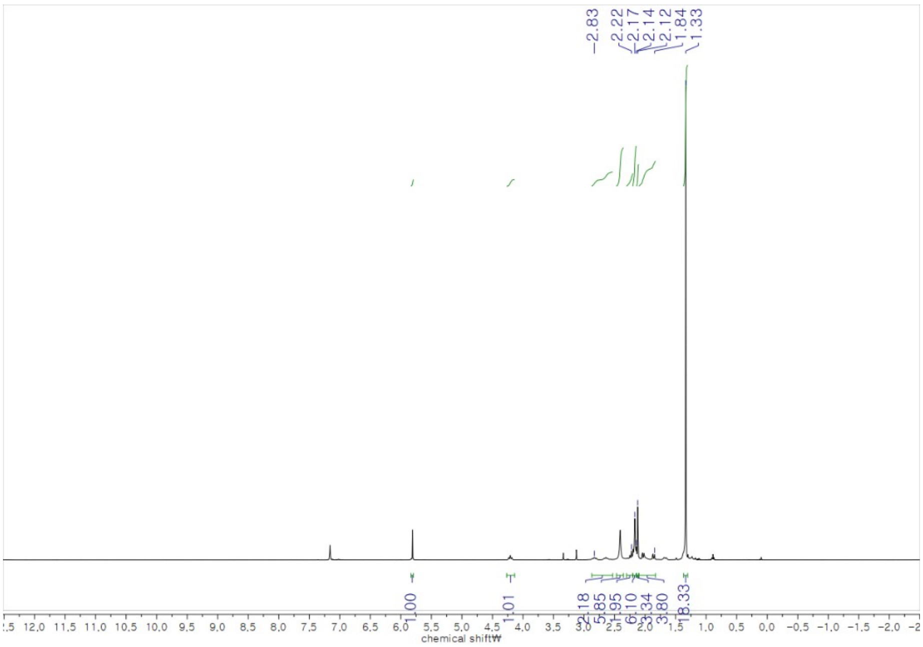

Figure S1. ^{1}H-NMR spectra of complexes **1.**

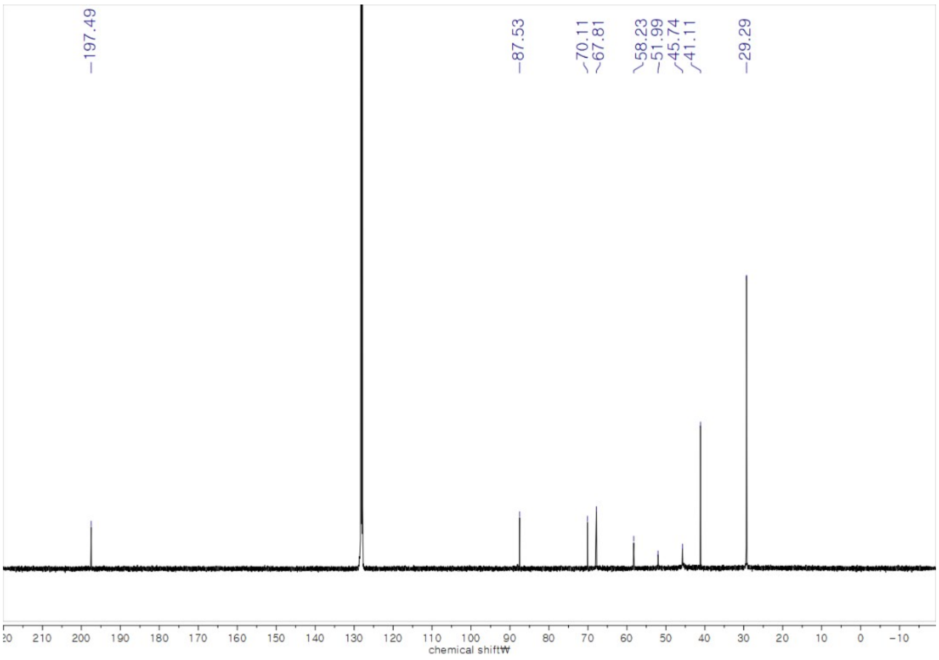

Figure S2. ^{13}C-NMR spectra of complex **1.**

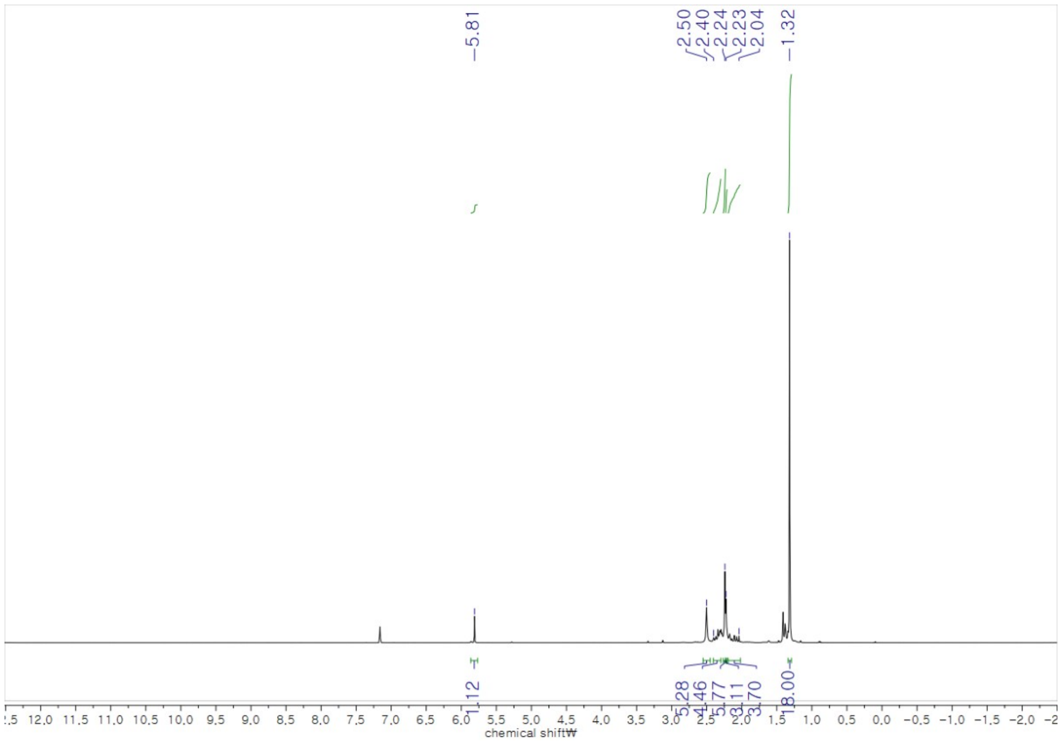

Figure S3. ^{1}H-NMR spectra of complex **2.**

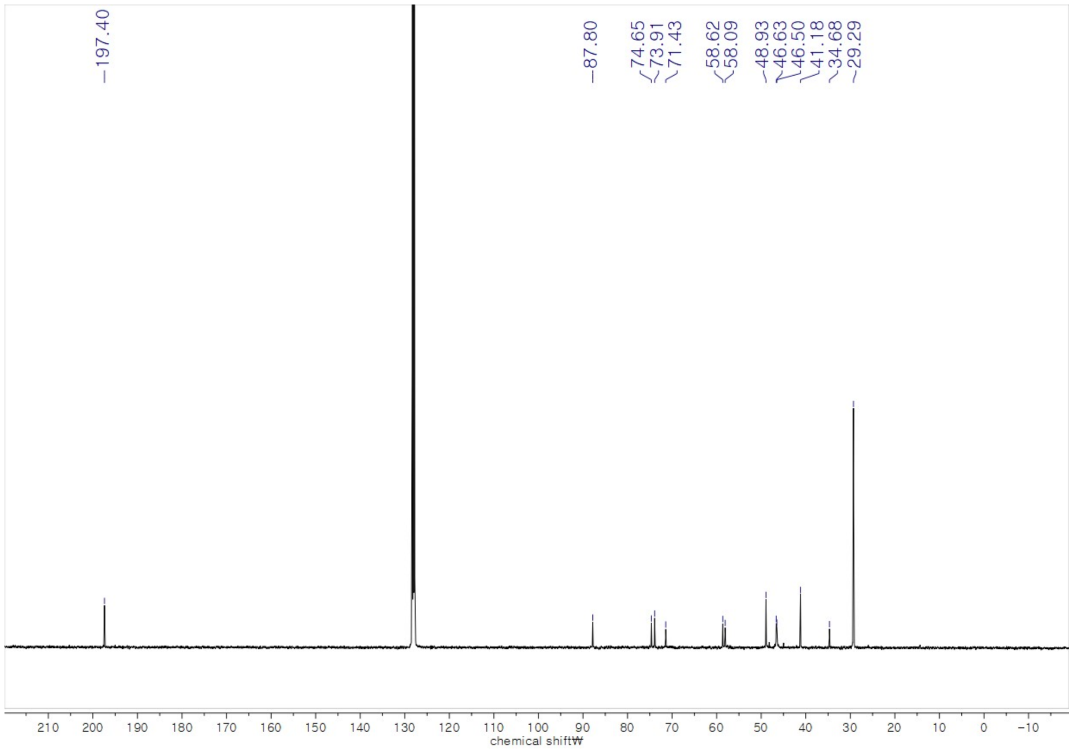

Figure S4. ^{13}C-NMR spectra of complex **2.**

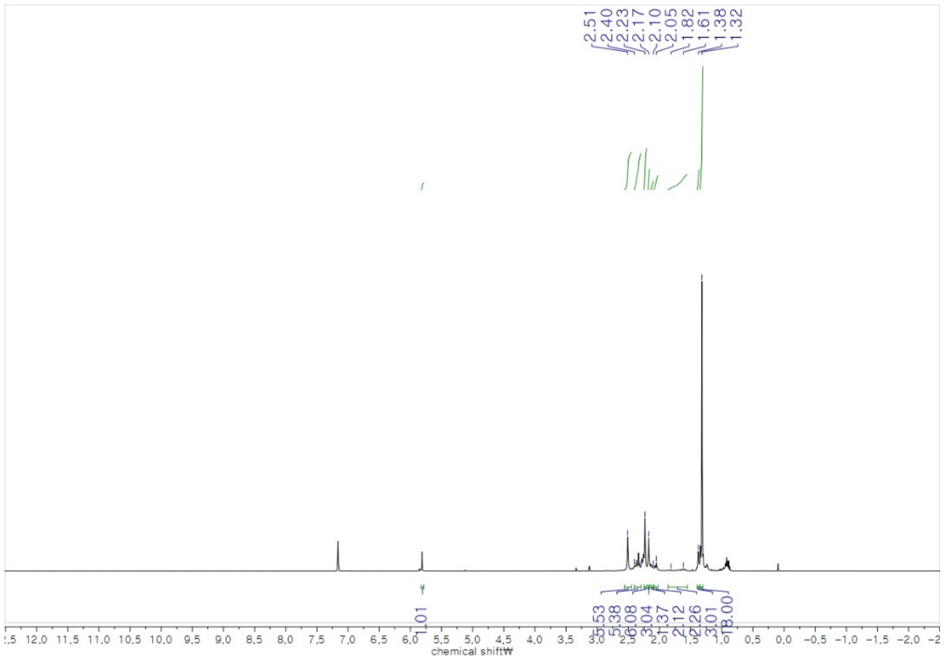

Figure S5. ^{1}H-NMR spectra of complex **3.**

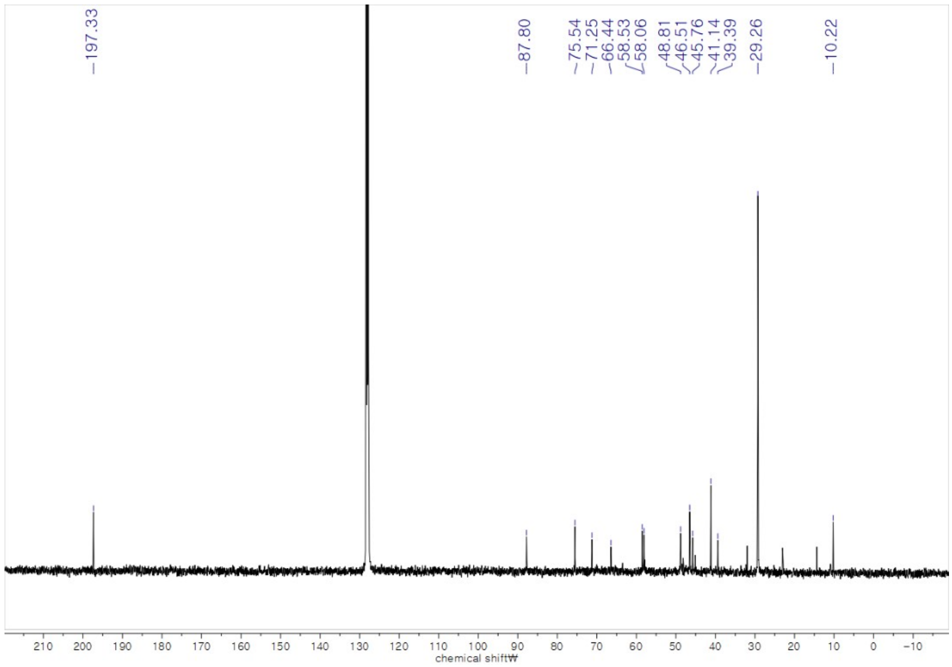

Figure S6. ^{13}C-NMR spectra of complex **3.**

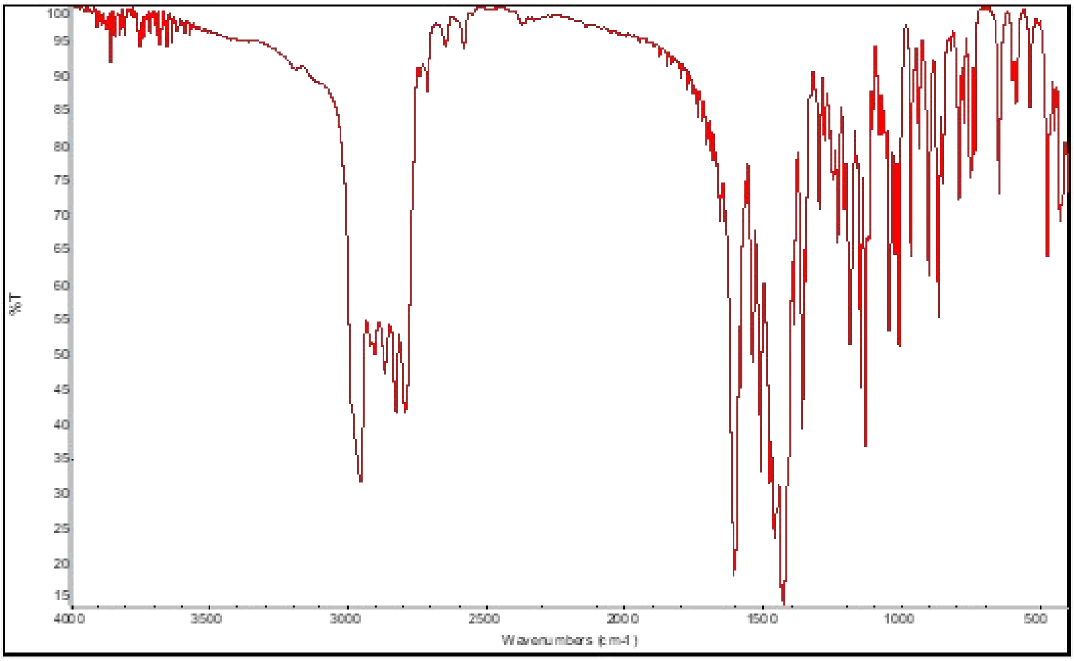

Figure S7. FT-IR spectra of complex **1.**

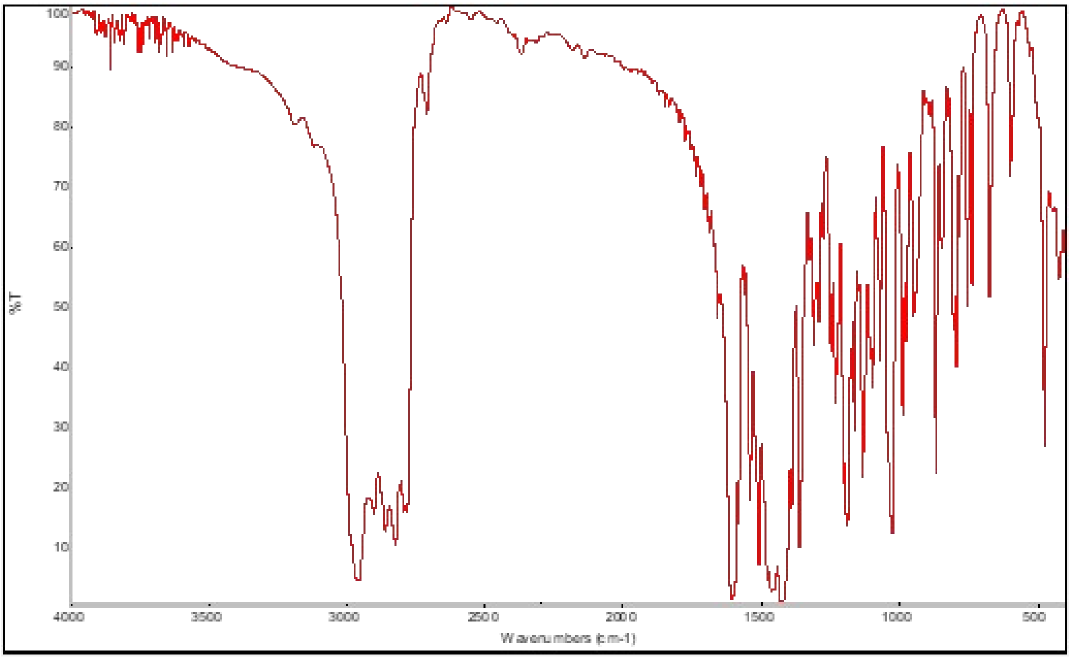

Figure S8. FT-IR spectra of complex **2.**

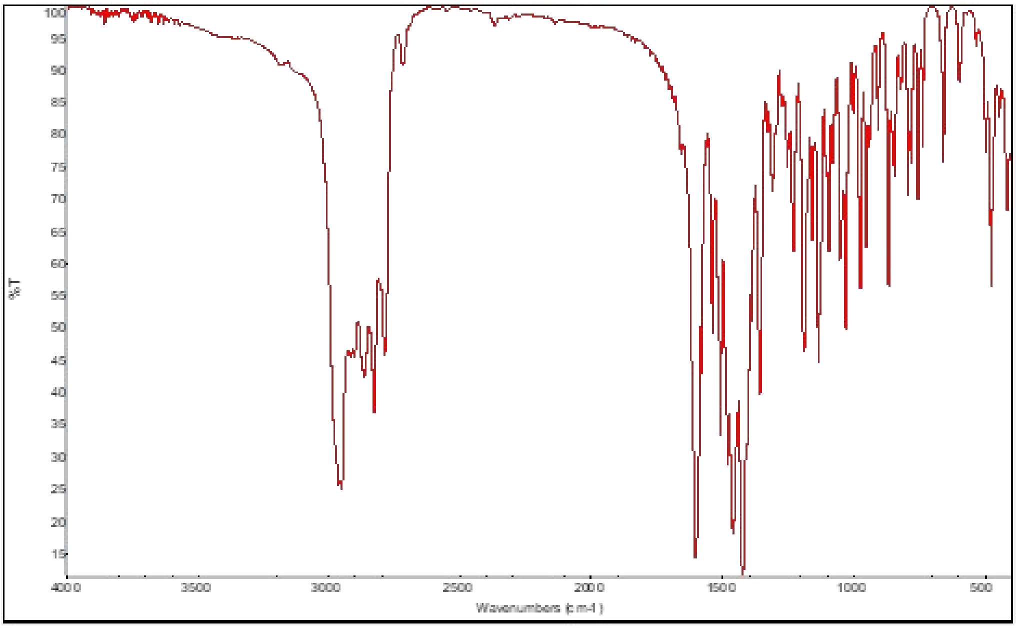

Figure S9. FT-IR spectra of complex **3.**

Table S1. X-ray data collection and structure refinement for complex **1**.

Compound		**1**	
Formula weight	2558.9(2)	Z	4
Temperature (K)	100	$\rho_{calcd.}$(Mg/m^3)	1.223
Wavelength (Å)	0.71073	μ (mm^{-1})	2.129
Crystal system	Monoclinic	F(000)	994.5
Space group	*P2(1)/n*	Crystal size(mm^3)	0.22X0.14X0.10
a (Å)	12.6947(7)	Theta range (°)	2.39 to 25.40
b (Å)	16.0289(8)	Index ranges	$-11 \leq h \leq 15$, $-17 \leq k \leq 19$, $-15 \leq l \leq 11$
c (Å)	12.9162(6)	Indep. refl.	32673
α (°)	90	Parameters	263
β (°)	103.190(2)	GOF on F^2	1.054
γ (°)	90	$R1$ [$I>2\sigma(I)$] (a)	0.0311
V (Å^3)	2558.9(2)	$wR2$ [$I>2\sigma(I)$] (b)	0.0855

Table S2. Selected bond lengths (Å) and bond angles (°) for complex **1**.

Complex **1**		Complex **2**	
	Bond Lengths (Å)		
Sr1-Sr1	3.884(4)	Sr1-O3(2)	2.4298(16)
Sr1-O1	2.4306(17)	Sr1-N5	2.8440(2)
Sr1-O2	2.4964(16)	Sr1-N6	2.7900(2)
Sr1-O3	2.4188(16)	Sr1-N7	2.7300(2)
	Bond Angles (°)		
O1 Sr1 O2	70.53(6)	N5 Sr1 O2	77.13(6)
O3 Sr1 O2	147.58(6)	N5 Sr1 O1	102.76(6)
O3 Sr1 O2	138.43(5)	N5 Sr1 O3	63.94(5)
O3 Sr1 O1	103.33(6)	N5 Sr1 O3	135.01(6)
O3 Sr1 O1	100.06(6)	N7 Sr1 O2	83.46(6)
N6 Sr1 O2	83.05(6)	N7 Sr1 O1	142.42(6)
N6 Sr1 O1	83.73(6)	N7 Sr1 O3	114.05(6)
N6 Sr1 O3	64.86(6)	N7 Sr1 O3	87.42(6)
N6 Sr1 O3	138.21(6)	N7 Sr1 N5	97.07(6)
N6 Sr1 N5	155.48(6)	Sr1 O1 Sr1(2)	106.47
